# Physicochemical and Genotoxic Evaluations of Singed Cowhide Meat (*Ponmo*) Wastewater

**DOI:** 10.5696/2156-9614-8.20.181207

**Published:** 2018-12-03

**Authors:** Ebenezer Olasunkanmi Dada, Hadijat Oluwatobi Osilagun, Kelechi Longinus Njoku

**Affiliations:** Department of Cell Biology and Genetics, Environmental Biology Unit, Faculty of Science, University of Lagos, Lagos, Nigeria

**Keywords:** allium assay, genotoxicity, *ponmo*, singed cow skin, wastewater

## Abstract

**Introduction.:**

In spite of doubts over the safety and nutritional benefits of singed cowhide meat, called *ponmo* in some parts of Nigeria, and its potential negative impact on the hide and leather industry, consumption in Nigeria and many parts of Africa has continued unabated.

**Objectives.:**

In the present study, physicochemical and genotoxic assessments of wastewater used to rinse ready-to-cook singed cowhide meat were carried out.

**Methods.:**

Physicochemical analyses were carried out using the American Public Health Association procedures, while genotoxic assessment was carried out using Allium cepa chromosome assay.

**Results.:**

The results of the physicochemical analyses indicated that some of the parameters, especially metals, were within the threshold of the limits set by the country's regulatory agencies, but some parameters like phosphate, chloride, nitrate, biological oxygen demand, and chemical oxygen demand were higher in concentration. The wastewater inhibited the growth of A. cepa roots and caused a decrease in its mitotic index relative to the control onions exposed to water only. The highest root growth inhibition of 72.14% was induced by a 10% wastewater concentration, while the lowest (53.57%) was induced by a 5% wastewater concentration. In addition, the wastewater induced bi-nucleated, attached, vagrant, C-mitosis, sticky and bridged chromosomal aberrations. Wastewater at a 5% concentration induced the highest significant (P < 0.05) percentage chromosome aberration of 36.62% at 48 hours of exposure. Sticky chromosomes had the highest significant frequency (P <0.01) at the end of the 72-hour exposure period. No chromosomal aberration was observed in the control.

**Conclusions.:**

These results indicate that singed cowhide meat wastewater is potentially genotoxic and environmentally harmful. Governments, public health practitioners, and relevant stakeholders should work in synergy to discourage the habit of processing cowhide into cowhide meat.

**Competing Interests.:**

The authors declare no competing financial interests.

## Introduction

Meat is a commonly consumed food worldwide.^1^ Preferences for a type of meat, methods of dehairing slaughtered animals, and of processing dehaired meat for consumption differ from country to country and from culture to culture. In Nigeria and many other parts of Africa, in addition to the regular consumption of red meat, the habit of eating dehaired cow skin is common.^2^ This is despite the fact that governments, concerned organizations and individuals have mounted campaigns against the habit, since it poses a potential danger to the health of consumers and potentially threatens the leather industry.^3^

In the southwestern part of Nigeria especially, dehaired cowhide meat is popularly referred to as *ponmo*. *Ponmo* is cowhide that has been processed, first by dehairing, to look like meat. There are two types; the first type is dehaired by shaving, called white *ponmo*, the other is dehaired by singeing, called brown *ponmo*. The names ‘white *ponmo*’ and ‘brown *ponmo*’ are reflections of their respective colors after dehairing and processing. Traditionally, singeing of cow skin to brown *ponmo* uses firewood for singeing, but due to a number of factors such as the unavailability of firewood, increasing number of cowhides for singeing, expanding *ponmo* market, urbanization, the urge to maximize profit etc, it became practically impossible for those in the *ponmo* business to continue to use firewood for singeing. As a result, various substances such as used plastics, scrapped tires, kerosene, and spent engine oil are used as fire sources, either alone or in combination with wood, to singe cowhide to brown *ponmo*.^2^,^4^,^5^ The singed cowhide is thereafter washed several times and then boiled in water for several hours to bring about the initial softening of the hide. The boiled, singed hide is subjected to final softening by soaking in water until it is tender enough for cooking and looks appealing to consumers. Cow skin or hides that are dehaired by singeing are potentially exposed to contaminants like toxic organic compounds (polyaromatic hydrocarbons, dioxins, furans, benzene) and trace/toxic metals which may be deposited by the singeing substrate.^4^ Studies have confirmed that cowhides processed for consumption by singeing have a higher contaminant load, especially metals, relative to those processed by other methods like shaving.^4^,^6^,^7^ In the present study, the wastewater in which singed cowhide meat had been soaked was first analyzed for physicochemical parameters, and thereafter tested for genotoxicity.

## Methods

**Figures 1 & 2:**
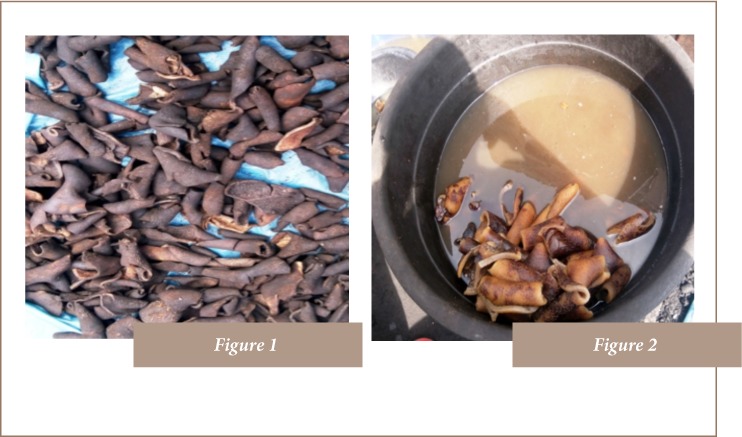
(Figure 1) Singed cowhide made ready for processing by cutting into pieces; (Figure 2) Pieces of processed, singed ready-to-cook cowhide soaking in wastewater

The common purple onion bulbs of average size (diameter: 5 to 6 cm) used in the present study were bought from Bariga market, Lagos (latitude 6°54′N and longitude 3°39′E). The wastewater in which singed cowhide meat received a final soak was obtained from a vendor at Ketu Market, in Lagos, Nigeria (latitude 6°58′N and longitude 3°34′E). A picture of singed cowhide made ready for processing by cutting into pieces is shown in Figure 1. Figure 2 shows pieces of processed, singed, ready-to-cook cowhide meat soaking in the wastewater used to for the present study.

### Physicochemical analyses of singed cowhide meat wastewater

The physicochemical analyses of the singed cowhide meat (*Ponmo*) wastewater were carried out in the laboratory of the Lagos State, Nigeria Environmental Protection Agency (LASEPA) using standard American Public Health Association(APHA) procedures as described in Okiei et al.^2^,^8^ The physicochemical properties tested included appearance, pH, total solids, total dissolved solids, dissolved oxygen (DO), 5-day biochemical oxygen demand (BOD5), chemical oxygen demand (COD), lead (Pb), zinc (Zn), cadmium (Cd), copper (Cu), and chromium (Cr).

**Abbreviations**
*BOD*: Biochemical oxygen demand
*COD*: Chemical oxygen demand

### Allium cepa genotoxicity test

The Allium assay test was adapted from Fiskesjo, Bakare et al., and Olorunfemi et al.^9^,^10^,^11^ The outer scales and brownish bottom plate of sun-dried onion bulbs were carefully removed, leaving the ring of primordial root intact. The peeled bulbs were placed in dechlorinated tap water during the cleaning procedure to prevent the primordial root from drying up.

The onion bulbs were grown in tap water at room temperature (25–30°C) for 24 hours. When the roots were 2–3 cm long, the bulbs were transferred to the *ponmo* wastewater of concentrations 5%, 10%, 20% and 100% (v/v, effluent/water). The test substrates were changed daily. Five onion bulbs were set up for each concentration including the control, out of which the best four were selected for evaluation. The root lengths of the onions were measured from 24 hours to 72 hours using a meter rule and expressed in centimeters (cm) as described by Fiskesjo.^10^ The mean root length, percentage root length, and percentage root length inhibition were calculated using Equations 1, 2, and 3 respectively.

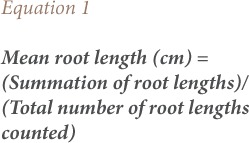


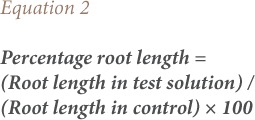


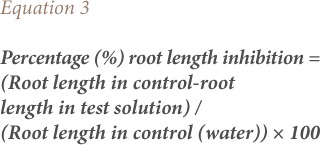



The squash technique for onion root as described by Adegbite and Olorode was used for the chromosomal investigation.^12^ Chromosome samples were taken from the root tip meristem containing actively growing cells. One root tip was squashed on each slide and stained with acetocarmine for 10 minutes. Cover slips were carefully lowered onto the slide to exclude air bubbles. To prevent the possible drying out of the preparation, the cover slips were sealed on the slides with clear fingernail polish. The slides were observed under the light microscope (Leica 2000 phase contrast microscope). Data on total cells, total dividing cells, and cells carrying chromosomal aberrations were taken from the slides prepared for each of the different concentrations and the control.

The mitotic index and percentage mitotic index were calculated using Equations 4 and 5.

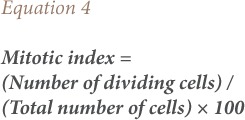


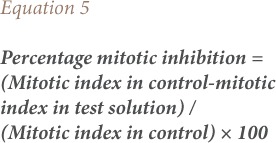



The frequency of chromosomal aberrations was calculated by expressing the number of aberrant cells as a percentage of total dividing cells for each treatment (*Equation 6*). Scoring of chromosomal aberrations was taken from five microscopic fields for each of the different test solutions.

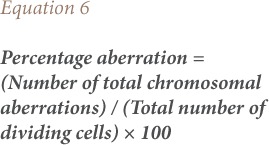



### Statistical data analyses

Data on the number of different aberrant chromosome types at the end of 72 hours were processed and compared by general linear model univariate analysis of variance. The mean values were compared for level of significance using the least significant difference test. All statistical analyses were carried out with IBM Statistical Package for the Social Sciences (SPSS) version 20 software.

## Results

The results of the physicochemical analyses carried out on the cowhide meat wastewater indicated that some of the parameters, especially trace/toxic metals, were below or within the threshold of the limits set by the country's regulatory agencies, Lagos State Environmental Protection Agency (LASEPA), Nigeria; and National Environmental Standards and Regulations Enforcement Agency (NESREA).^13^ However, some chemical parameters like phosphate, chloride, nitrate, BOD, and COD were higher in concentration (*Table 1*).

**Table 1:**
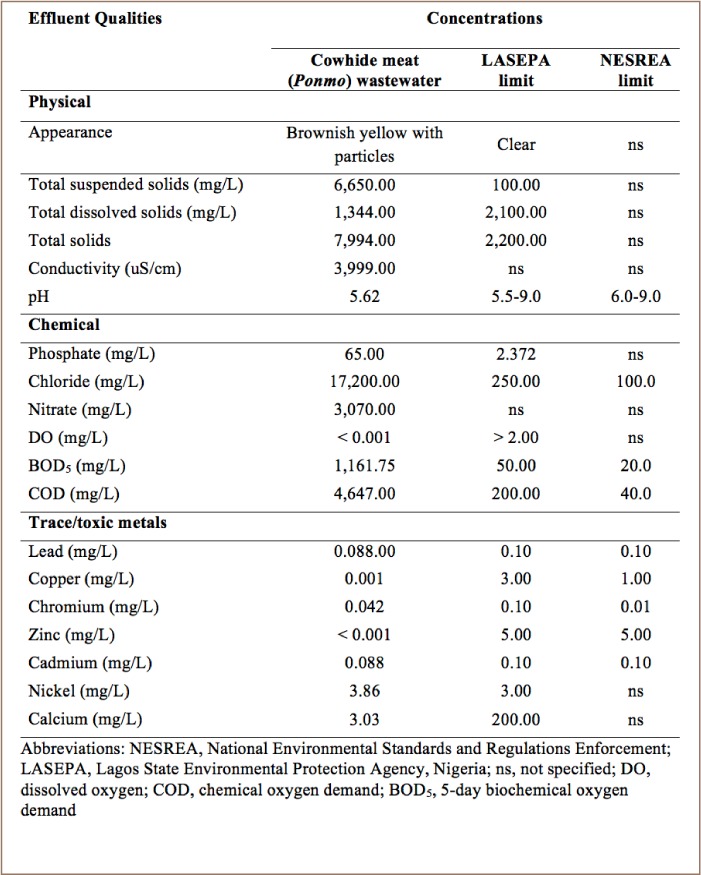
Physicochemical Qualities of Cowhide Meat (Ponmo) Wastewater

### Effect of cowhide meat wastewater on the root growth of A. cepa

Cowhide wastewater inhibited the growth of A. cepa roots (*Table 2*). The highest root growth inhibitions produced by 5%, 10%, 20% and 100% *ponmo* effluent occurred at 72 hours of exposure. The highest root growth inhibition of 72.14% was induced by 10% wastewater concentration, while the lowest (53.57%) was induced by 5% wastewater concentration.

**Table 2:**
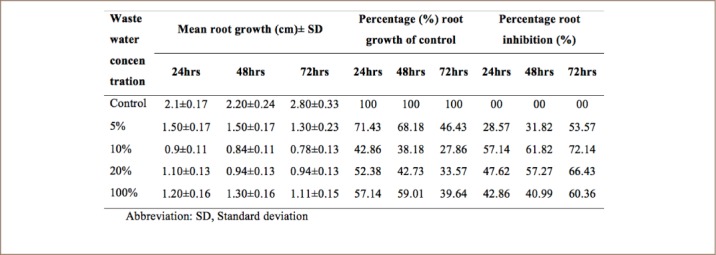
Effects of Cowhide Meat (Ponmo) Wastewater on the Root Growth of A. cepa

### Mitotic activity and mitotic inhibition induced in A. cepa exposed to cowhide meat wastewater

The mitotic activity in the roots of A. cepa exposed to *ponmo* wastewater indicated that the wastewater caused a decrease in the mitotic index relative to the control onions exposed to water only. While the highest mitotic index (50%) was recorded in control onions exposed to water only, the lowest (3.33%) was recorded in the roots of onions exposed to a 100% *ponmo* wastewater concentration. No mitotic inhibition was recorded in onions exposed to water only, while the highest mitotic inhibition (93.34%) was recorded in those exposed to a 100% wastewater concentration. It was observed that the highest mitotic inhibitions induced by 5%, 10%, 20% and 100% *ponmo* wastewater concentrations all occurred at 72 hours of exposure (*Table 3*).

**Table 3:**
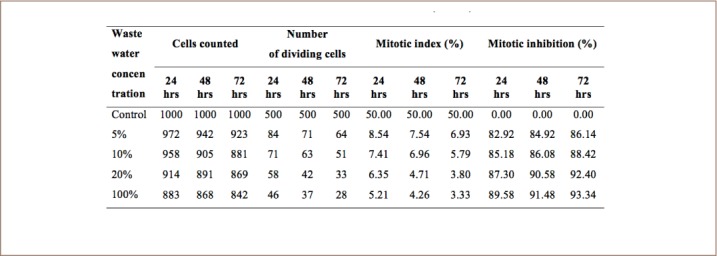
Mitotic Index and Mitotic Inhibitions in A. cepa Exposed to Different Concentrations of Cowhide Meat (Ponmo) Wastewater

### Chromosome aberrations induced by cow skin meat wastewater

**Figures 3–8:**
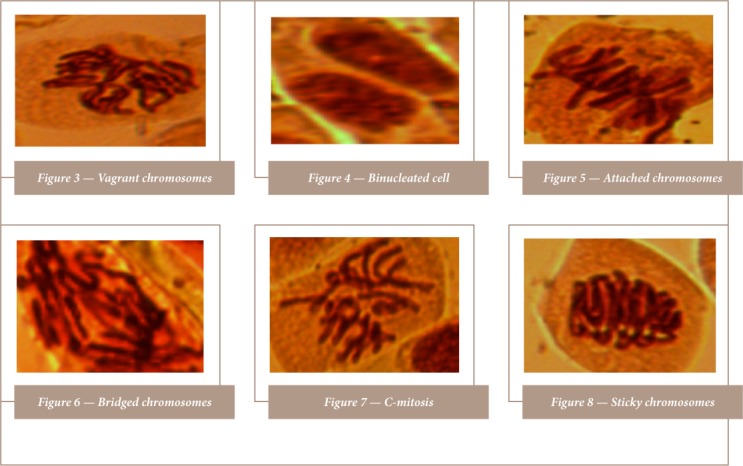
Representative samples of aberrant chromosomes induced by singed cowhide meat (ponmo) wastewater

Cow skin meat wastewater induced chromosome aberrations in the roots of A. cepa (*Table 4*). No chromosome aberration was observed in onions exposed to water (control). The chromosome aberrations observed were bi-nucleated, attached, vagrant, C-mitosis, sticky and bridged chromosomes. At 24 and 48 hours of exposure, chromosome aberrations were highest in 5% *ponmo* wastewater, while at 72 hours, chromosome aberrations were highest in 10% wastewater. Sticky chromosomes had the highest significant frequency (P <0.01) at the end of the 72-hour exposure period. Representative samples of aberrant chromosomes are shown in Figures 3 to 8.

**Table 4:**
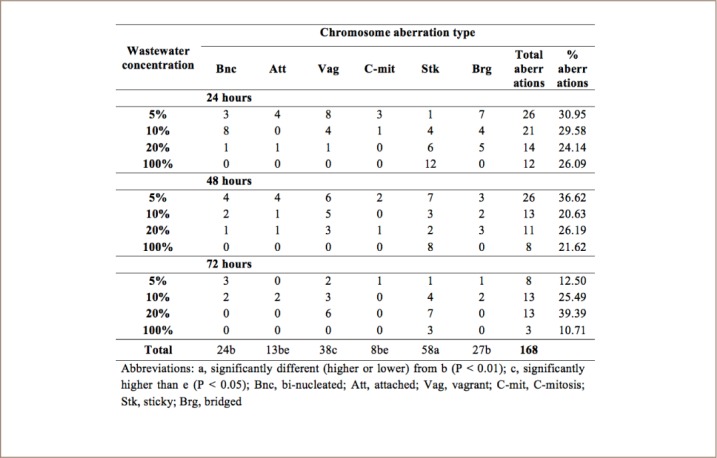
Chromosome Aberrations Induced in A. cepa Root Cells Exposed to Cowhide Meat (Ponmo) Wastewater

## Discussion

Results from the present study revealed that some physicochemical properties, especially metal concentrations, of the cowhide meat wastewater used in this study were within the threshold of the limits set by the Nigerian regulatory agencies.^13^ However, many parameters like phosphate, chloride, nitrate, BOD, and COD were higher in concentration. The high level of total suspended solids most likely resulted from the crumbs of processed cowhide meat as evidenced by the appearance of the wastewater, which was brownish yellow with suspended particles. In addition, the organic nature of cowhide meat and its crumbs were likely responsible for the elevated levels of COD and BOD in the wastewater.^14^ The high BOD level was also likely responsible for the very low DO in the cowhide meat wastewater as the presence of high BOD levels in a water body will always bring about accelerated bacterial growth accompanied by correspondingly high oxygen consumption.^15^ Although past studies that directly measured the quality of cowhide wastewater could not be found in the literature search, other studies have confirmed that cowhide processed into meat by singeing are particularly prone to harboring relatively higher levels of metals and other contaminants deposited by the various substances used as a flame treatment.^2^,^4^,^16^ It can therefore be assumed that the bulk of the metal loads observed in the wastewater was released by the singed cowhide meat.

In general, the operators of cowhide meat businesses dispose of their wastewater in drainage canals that eventually empty into the lagoon, thereby increasing the pollution load of lagoon water and resident organisms like fish, prawns, and crabs, some of which serve as food for humans. A persistently low DO in a water body may be lethal for most fish and other aquatic animals, while chronic and cumulative exposure to metals like Cd, Cu, Pb may cause a variety of problems including disruption in normal vitamin uptake. Cadmium is a cumulative contaminant that is toxic to both humans and fish.^15^ Excessive suspended solids in aquatic habitats can contribute to increased turbidity leading to reduced light penetration and primary productivity.^17^ This suggests that singed cowhide meat (brown *ponmo*) is not only potentially harmful to the health of those who consume it, but the wastewater used to process it also constitutes a possible pollution threat to the environment, especially the aquatic environment.^2^,^4^,^16^ Therefore, the discharge of this wastewater directly into waterways should be addressed.

The present study did not assess cowhide meat genotoxicity directly, rather, it evaluated the wastewater used in its processing as the A. cepa assay requires a liquid medium. Not only did the wastewater inhibit A. cepa root growth, it was also mitodepressive and genotoxic, inducing chromosomal aberrations such as binucleated, attached, C-mitosis, sticky and bridged chromosomes. This lends further credence to the assumption that singed cowhide meat wastewater is potentially harmful to the aquatic environment. The decreased mitotic index and the chromosomal aberrations produced in A. cepa roots were likely due to the presence of metals and other parameters in the wastewater. The decreased mitotic index is believed to result from either a disturbance in the cell cycle or chromatin dysfunction induced by metal-DNA interactions.^18^ Sticky chromosomes, which were the most frequent aberrations recorded, indicate disruption of chromatin organization by contaminants.^19^ Sticky chromosomes have been considered a common sign of chromosomal toxicity that may lead to cell death.^20^ Since studies have correlated chromosome abnormalities and mutagenic activity found in root-tip systems with those found in mammalian cell systems, the genotoxic effects produced by cowhide meat wastewater in the root cells of A. cepa in the present study can be extended to mammals.^13^,^21^ Since the effects of cowhide meat wastewater observed in this study were likely induced by the singed cowhide itself, the physicochemical characteristics and genotoxic effects of the wastewater observed in this study can equally be considered indirect effects of singed cowhide meat. Also worthy of note is the fact that singeing cowhide with wood, used tires, spent engine oil and other materials, as practiced by *ponmo* operators presents potential air pollution problems.

## Conclusions

The results of the present study indicate that singed cowhide meat wastewater is potentially genotoxic and environmentally harmful. Governments, public health practitioners, and relevant stakeholders should work in synergy to discourage the habit of processing cowhide into cowhide meat. This will help to protect human health, block a potential environmental pollution source, and at the same time save the hide and leather industry from the indirect competition it currently faces from cowhide meat.
